# Supplementary material for: Epidemiology and associated microbiota changes in deployed military personnel at high risk of traveler's diarrhea
Source: PLoS One. 2020 Aug 12;15(8):e0236703. doi: 10.1371/journal.pone.0236703 (PMC7423091; doi:10.1371/journal.pone.0236703)
Supplement: S1 File — (PDF) [file pone.0236703.s002.pdf]

SELF-REPORTED DEPLOYMENT SURVEY

The purpose of this survey is to understand the burden of various self-reported illnesses that impact our force readiness while deployed. Your participation in this questionnaire is completely anonymous and any information you give is strictly confidential. No data will be reported at the individual level. Your participation is greatly appreciated.

1. Today's date: _ _ / _ _ / _ _ _ _ (mm/dd/yyyy) 2. Age: __ __ 3. Sex: ☐ M ☐ F

3. Which branch of service are you currently in?
   a. ☐Air Force b. ☐Army c. ☐Marine Corps
   d. ☐Navy e. ☐ Civilian/Other

4. Grade/Rank:
   a. ☐E1-E3 b.☐E4-E6 c.☐E7-E9 d.☐ Warrant
   e. ☐ O1-O3 f. ☐O4-O5 g.☐O6 and above

5. Military Component:
   A. ☐Regular B. ☐Reserve C. ☐ National Guard
   D. ☐ Other:___________

6. Date deployed to current location:
   _ _ / _ _ / _ _ _ _ (mm/dd/yyyy) (mm/yyyy) if day not known

7. Anticipated date of departure from current location:
   _ _ / _ _ / _ _ _ _ (mm/dd/yyyy) (mm/yyyy) if day not known ☐ Don't Know

8. Have you been to any other countries (excluding the US) for more than 24 hours during this deployment? If cannot state country, state region (Central America, South American, W Africa, etc) Yes No **If no, go to question 9.**

   a. **If yes, list countries/regions starting with the most recent?**

   Country/Region #1:
   Month/year of travel:(mm/yyyy) _ _ / _ _ _ _ # of days in country _ _
   Country/Region #2:
   Month/year of travel:(mm/yyyy) _ _ / _ _ _ _ # of days in country _ _
   Country/Region #3:
   Month/year of travel:(mm/yyyy) _ _ / _ _ _ _ # of days in country _ _
   Country/Region #4:
   Month/year of travel:(mm/yyyy) _ _ / _ _ _ _ # of days in country _ _

   Please list any additional countries where you stayed at least 24 hours:____

Diarrhea Survey: PLEASE REPORT ILLNESSES DURING YOUR DEPLOYMENT

9. Have you had diarrhea? (3 or more loose or watery stools during any 24-hour period or 2 or more stools with additional symptoms such as bloody stool cramping, nausea, vomiting, fever, headache. A typical diarrhea episode lasts 2 to 5 days)
   Yes No **If no, skip to Q16 on the next page (other side).**

10. Enter the number of diarrhea episodes during this deployment. _ _

11. Do you currently have diarrhea? Yes No If **yes**, how many days? _ _

12. **Fill out the following chart, recording the three most severe episodes of diarrhea starting with the most recent.**

a. **Approximate date of Episode**	**(mm/yyyy)** _ _/_ _ _ _	**(mm/yyyy)** _ _/_ _ _ _	**(mm/yyyy)** _ _/_ _ _ _
b. Did you have any vomiting?	Yes No<br>If yes, # of episodes: _ _	Yes No<br>If yes, # of episodes: _ _	Yes No<br>If yes, # of episodes: _ _
c. Did you have a fever or feel feverish?	Yes No	Yes No	Yes No
d. Was there blood in the diarrhea?	Yes No	Yes No	Yes No
e. Were there muscle/joint aches with this episode?	Yes No	Yes No	Yes No
f. Did you have nausea?	Yes No	Yes No	Yes No
g. Did you have a headache?	Yes No	Yes No	Yes No
h. Did you have abdomen cramps?	Yes No	Yes No	Yes No
i. Maximum # of stools in a 24 hour period?	_ _	_ _	_ _
j. How many days did the episode of diarrhea last?	_ _(Days)	_ _(Days)	_ _(Days)
k. How many days of work did you miss due to this episode?	_ _(Days)	_ _(Days)	_ _(Days)
l. How many days of decreased performance?	_ _(Days)	_ _(Days)	_ _(Days)
m. Did you travel off the ship within the last 14 days before episode started?	Yes No<br>If yes, where__________	Yes No<br>If yes, where__________	Yes No<br>If yes, where__________
n. Were you put on bed-rest or SIQ for this episode?	Yes No	Yes No	Yes No
o. Did you go to sick call/clinic for this episode?	Yes No	Yes No	Yes No
p. Did you receive intravenous fluids for this episode?<br>If yes, approximate # of liters.	Yes No<br>_ _(# of liters)	Yes No<br>_ _(# of liters)	Yes No<br>_ _(# of liters)
q. Who treated this episode? Check all that apply<br>**(If no treatment, go to Q13)**	☐Self ☐ Doctor<br>☐Medic ☐None	☐Self ☐ Doctor<br>☐Medic ☐None	☐Self ☐ Doctor<br>☐Medic ☐None
r. What was the regimen/medication and how did you receive it? Mark "**S**" for self, "**P**" for provider, or "**SP**" for both.	☐Antibiotics ☐Imodium<br>☐Pepto ☐Other_______	☐Antibiotics ☐Imodium<br>☐Pepto ☐Other_______	☐Antibiotics ☐Imodium<br>☐Pepto ☐Other_______

PLEASE CONTINUE TO OTHER SIDE

13. Were you given or did you bring antibiotics or other antidiarrheal medications prior to travel/deployment in case you need them during travel/deployment? Yes No

14. Have you eaten off base in the past 14 days? Yes No

15. Were back-up personnel required to come in to cover you while you were sick? Yes No

16. Did a diarrheal episode result in you missing an important mission/assignment? Yes No

17. In a combat situation, would this illness affect your ability to perform your duties? Yes No

Respiratory Illness Survey

18. Provide the number of respiratory illnesses (symptoms of fever plus cough and/or sore throat) did you have during this deployment? (Enter 00 for none) _ _ **If "none", go to Question 27.**

19. Approximate dates(mm/yyyy) and durations (in days) for each episode of illness:

#1: _ _ / _ _ _ _; _ _(days) #2: _ _ / _ _ _ _; _ _(days) #3: _ _ / _ _ _ _; _ _(days) #4: _ _ / _ _ _ _; _ _(days)

If you had more than one episode, answer the following questions for the most severe episode. If only one, answer for that single episode.

20. Circle any related symptoms (for the most severe episode):

a.	Fever/feverish	Yes	No	f.	Headache?	Yes	No
b.	Diarrhea	Yes	No	g.	Conjunctivitis (Pink Eye)	Yes	No
c.	Vomiting	Yes	No	h.	Muscle/joint aches	Yes	No
d.	Night sweats	Yes	No	i.	Shortness of breath	Yes	No
e.	Cough	Yes	No	j.	Sore throat	Yes	No

21. Were you evaluated for this episode in sick call?
Yes No **If yes, what was the diagnosis?**
____

22. Were you hospitalized for this illness?
Yes No
a. If yes, enter the number of days: _ _

23. Were you put on SIQ/bed-rest because of this illness?
Yes No
a. If yes, enter number of days: _ _

24. Did having an episode of illness decrease your ability to work? Yes No

25. Did you travel off the ship within the last 14 days before episodes started? Yes No
a. If yes, where? ___________________

26. How was the most severe episode treated?
None Self Doctor Medic
Medication:______________________

27. Did you receive Influenza vaccine within the last 12 months?
Yes No

28. Which antimalarial medication have you been on most while deployed?

A. Chloroquine B. Doxycycline C. Mefloquine(Lariam)
D. Malarone E. Primaquine F. Other:__________

Acute Febrile Illness

29. Enter number of times you had an illness with fever but not associated with diarrhea or respiratory symptoms or other obvious cause (such as ear infection, skin infection, etc)? (Enter 0 for none) _ _ **If none, end of survey.**
a. Approximate dates(mm/yyyy) and duration (days) for each episode of illness:
#1: _ _ / _ _ _ _; _ _(days) #2: _ _ / _ _ _ _; _ _(days)
#3: _ _ / _ _ _ _; _ _(days) #4: _ _ / _ _ _ _; _ _(days)

If you had more than one episode, answer below for the most severe episode. If only one, answer for the single episode:

30. Did you have any other symptoms related to this illness?
Yes No **If yes, list**:___________________________

31. Were you evaluated for this episode in sick call?
Yes No **If yes, what was the diagnosis?**
____

32. Did you receive any treatment for this illness? Yes No
a. **If yes, list medication**:____________________

33. Did having an illness episode result decrease your ability to perform duties? Yes No

34. Did you travel off the ship within the last 14 days before episodes started? Yes No
a. If yes, where? ___________________

END OF SURVEY. THANK YOU FOR YOUR TIME.
