# Supplementary figures and images for: Epidemiology and associated microbiota changes in deployed military personnel at high risk of traveler's diarrhea
Source: PLoS One. 2020 Aug 12;15(8):e0236703. doi: 10.1371/journal.pone.0236703 (PMC7423091; doi:10.1371/journal.pone.0236703)

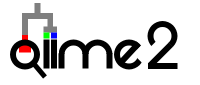

Supplement: S2 File — This includes per-sample metadata, and can be viewed at https://view.qiime2.org/. For example, choose taxonomic level 3 to see the class level, and under “Sort Samples By” select “Subject”, then click + to add additional sorting for “Order” and again for “ClinicalTD”. Taxonomies can be toggled by clicking the colored box next to the taxa. (QZV) [file pone.0236703.s003.qzv › fe6ac182-7d58-41eb-9744-6e2c31fcc5cc/data/q2templateassets/img/qiime2-rect-200.png]

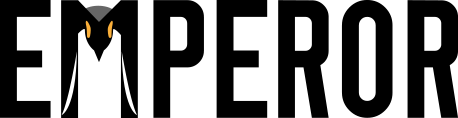

Supplement: S3 File — This can be viewed at https://view.qiime2.org/. For example, to view the data by TD subject, select “Subject” under the scatter dropdown box. Successive time points can be connected by clicking the animations tab, selecting Gradient->Order, Trajectory->Subject, and clicking the play button. (QZV) [file pone.0236703.s004.qzv › 7754985f-4143-4ce5-b961-22dcbcc4e402/data/img/emperor.png]

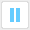

Supplement: S3 File — This can be viewed at https://view.qiime2.org/. For example, to view the data by TD subject, select “Subject” under the scatter dropdown box. Successive time points can be connected by clicking the animations tab, selecting Gradient->Order, Trajectory->Subject, and clicking the play button. (QZV) [file pone.0236703.s004.qzv › 7754985f-4143-4ce5-b961-22dcbcc4e402/data/img/pause.png]

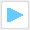

Supplement: S3 File — This can be viewed at https://view.qiime2.org/. For example, to view the data by TD subject, select “Subject” under the scatter dropdown box. Successive time points can be connected by clicking the animations tab, selecting Gradient->Order, Trajectory->Subject, and clicking the play button. (QZV) [file pone.0236703.s004.qzv › 7754985f-4143-4ce5-b961-22dcbcc4e402/data/img/play.png]

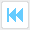

Supplement: S3 File — This can be viewed at https://view.qiime2.org/. For example, to view the data by TD subject, select “Subject” under the scatter dropdown box. Successive time points can be connected by clicking the animations tab, selecting Gradient->Order, Trajectory->Subject, and clicking the play button. (QZV) [file pone.0236703.s004.qzv › 7754985f-4143-4ce5-b961-22dcbcc4e402/data/img/reset.png]

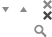

Supplement: S3 File — This can be viewed at https://view.qiime2.org/. For example, to view the data by TD subject, select “Subject” under the scatter dropdown box. Successive time points can be connected by clicking the animations tab, selecting Gradient->Order, Trajectory->Subject, and clicking the play button. (QZV) [file pone.0236703.s004.qzv › 7754985f-4143-4ce5-b961-22dcbcc4e402/data/vendor/css/chosen-sprite.png]

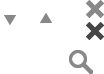

Supplement: S3 File — This can be viewed at https://view.qiime2.org/. For example, to view the data by TD subject, select “Subject” under the scatter dropdown box. Successive time points can be connected by clicking the animations tab, selecting Gradient->Order, Trajectory->Subject, and clicking the play button. (QZV) [file pone.0236703.s004.qzv › 7754985f-4143-4ce5-b961-22dcbcc4e402/data/vendor/css/chosen-sprite@2x.png]

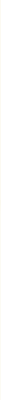

Supplement: S3 File — This can be viewed at https://view.qiime2.org/. For example, to view the data by TD subject, select “Subject” under the scatter dropdown box. Successive time points can be connected by clicking the animations tab, selecting Gradient->Order, Trajectory->Subject, and clicking the play button. (QZV) [file pone.0236703.s004.qzv › 7754985f-4143-4ce5-b961-22dcbcc4e402/data/vendor/css/images/ui-bg_glass_55_fbf9ee_1x400.png]

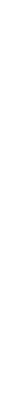

Supplement: S3 File — This can be viewed at https://view.qiime2.org/. For example, to view the data by TD subject, select “Subject” under the scatter dropdown box. Successive time points can be connected by clicking the animations tab, selecting Gradient->Order, Trajectory->Subject, and clicking the play button. (QZV) [file pone.0236703.s004.qzv › 7754985f-4143-4ce5-b961-22dcbcc4e402/data/vendor/css/images/ui-bg_glass_65_ffffff_1x400.png]

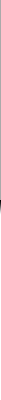

Supplement: S3 File — This can be viewed at https://view.qiime2.org/. For example, to view the data by TD subject, select “Subject” under the scatter dropdown box. Successive time points can be connected by clicking the animations tab, selecting Gradient->Order, Trajectory->Subject, and clicking the play button. (QZV) [file pone.0236703.s004.qzv › 7754985f-4143-4ce5-b961-22dcbcc4e402/data/vendor/css/images/ui-bg_glass_75_dadada_1x400.png]

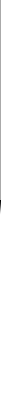

Supplement: S3 File — This can be viewed at https://view.qiime2.org/. For example, to view the data by TD subject, select “Subject” under the scatter dropdown box. Successive time points can be connected by clicking the animations tab, selecting Gradient->Order, Trajectory->Subject, and clicking the play button. (QZV) [file pone.0236703.s004.qzv › 7754985f-4143-4ce5-b961-22dcbcc4e402/data/vendor/css/images/ui-bg_glass_75_e6e6e6_1x400.png]

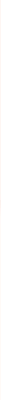

Supplement: S3 File — This can be viewed at https://view.qiime2.org/. For example, to view the data by TD subject, select “Subject” under the scatter dropdown box. Successive time points can be connected by clicking the animations tab, selecting Gradient->Order, Trajectory->Subject, and clicking the play button. (QZV) [file pone.0236703.s004.qzv › 7754985f-4143-4ce5-b961-22dcbcc4e402/data/vendor/css/images/ui-bg_glass_95_fef1ec_1x400.png]

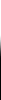

Supplement: S3 File — This can be viewed at https://view.qiime2.org/. For example, to view the data by TD subject, select “Subject” under the scatter dropdown box. Successive time points can be connected by clicking the animations tab, selecting Gradient->Order, Trajectory->Subject, and clicking the play button. (QZV) [file pone.0236703.s004.qzv › 7754985f-4143-4ce5-b961-22dcbcc4e402/data/vendor/css/images/ui-bg_highlight-soft_75_cccccc_1x100.png]

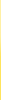

Supplement: S3 File — This can be viewed at https://view.qiime2.org/. For example, to view the data by TD subject, select “Subject” under the scatter dropdown box. Successive time points can be connected by clicking the animations tab, selecting Gradient->Order, Trajectory->Subject, and clicking the play button. (QZV) [file pone.0236703.s004.qzv › 7754985f-4143-4ce5-b961-22dcbcc4e402/data/vendor/css/images/ui-bg_highlight-soft_75_ffe45c_1x100.png]

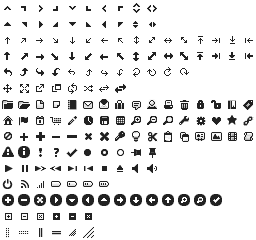

Supplement: S3 File — This can be viewed at https://view.qiime2.org/. For example, to view the data by TD subject, select “Subject” under the scatter dropdown box. Successive time points can be connected by clicking the animations tab, selecting Gradient->Order, Trajectory->Subject, and clicking the play button. (QZV) [file pone.0236703.s004.qzv › 7754985f-4143-4ce5-b961-22dcbcc4e402/data/vendor/css/images/ui-icons_222222_256x240.png]

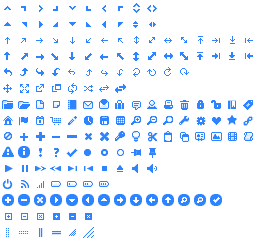

Supplement: S3 File — This can be viewed at https://view.qiime2.org/. For example, to view the data by TD subject, select “Subject” under the scatter dropdown box. Successive time points can be connected by clicking the animations tab, selecting Gradient->Order, Trajectory->Subject, and clicking the play button. (QZV) [file pone.0236703.s004.qzv › 7754985f-4143-4ce5-b961-22dcbcc4e402/data/vendor/css/images/ui-icons_2e83ff_256x240.png]

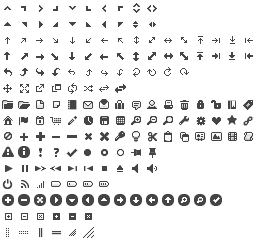

Supplement: S3 File — This can be viewed at https://view.qiime2.org/. For example, to view the data by TD subject, select “Subject” under the scatter dropdown box. Successive time points can be connected by clicking the animations tab, selecting Gradient->Order, Trajectory->Subject, and clicking the play button. (QZV) [file pone.0236703.s004.qzv › 7754985f-4143-4ce5-b961-22dcbcc4e402/data/vendor/css/images/ui-icons_454545_256x240.png]

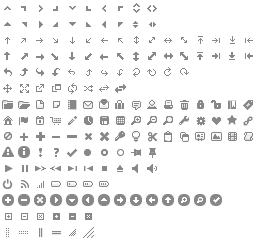

Supplement: S3 File — This can be viewed at https://view.qiime2.org/. For example, to view the data by TD subject, select “Subject” under the scatter dropdown box. Successive time points can be connected by clicking the animations tab, selecting Gradient->Order, Trajectory->Subject, and clicking the play button. (QZV) [file pone.0236703.s004.qzv › 7754985f-4143-4ce5-b961-22dcbcc4e402/data/vendor/css/images/ui-icons_888888_256x240.png]

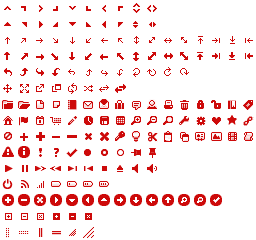

Supplement: S3 File — This can be viewed at https://view.qiime2.org/. For example, to view the data by TD subject, select “Subject” under the scatter dropdown box. Successive time points can be connected by clicking the animations tab, selecting Gradient->Order, Trajectory->Subject, and clicking the play button. (QZV) [file pone.0236703.s004.qzv › 7754985f-4143-4ce5-b961-22dcbcc4e402/data/vendor/css/images/ui-icons_cd0a0a_256x240.png]
