# Supplementary material for: Epidemiology and associated microbiota changes in deployed military personnel at high risk of traveler's diarrhea
Source: PLoS One. 2020 Aug 12;15(8):e0236703. doi: 10.1371/journal.pone.0236703 (PMC7423091; doi:10.1371/journal.pone.0236703)
Supplement: S3 File — This can be viewed at https://view.qiime2.org/. For example, to view the data by TD subject, select “Subject” under the scatter dropdown box. Successive time points can be connected by clicking the animations tab, selecting Gradient->Order, Trajectory->Subject, and clicking the play button. (QZV) [file pone.0236703.s004.qzv › 7754985f-4143-4ce5-b961-22dcbcc4e402/data/templates/standalone-template.html]

Emperor


{% include style\_template\_path %}

{% include logic\_template\_path %}
