# Supplementary material for: Epidemiology and associated microbiota changes in deployed military personnel at high risk of traveler's diarrhea
Source: PLoS One. 2020 Aug 12;15(8):e0236703. doi: 10.1371/journal.pone.0236703 (PMC7423091; doi:10.1371/journal.pone.0236703)
Supplement: Attachment — Submitted filename: Second_Revision_Response_to_reviewers.docx [file pone.0236703.s012.docx]

**Dear Editor and Reviewers,**

**We thank the Editor and Reviewers for their careful review of our manuscript and insightful comments. Our responses to the issues raised by the reviewers are listed following each of their comments below. Our responses are shown in bold.**

| **Date:** | Jul 01 2020 01:00PM |
| --- | --- |
| **To:** | "Michael Goodson" michael.goodson.4@us.af.mil |
| **From:** | "PLOS ONE" plosone@plos.org |
| **Subject:** | PLOS ONE Decision: Revision required [PONE-D-19-31202R1] |

PONE-D-19-31202R1
Epidemiology and associated microbiota changes in deployed military personnel at high risk of traveler's diarrhea
PLOS ONE

Dear Dr. Goodson,

Thank you for submitting your manuscript to PLOS ONE. After careful consideration, we feel that it has merit but does not fully meet PLOS ONE’s publication criteria as it currently stands. Therefore, we invite you to submit a revised version of the manuscript that addresses the points raised during the review process.
All four reviewers and I are in agreement that the manuscript is much improved. Reviewers 2 and 4 have a few minor, helpful suggestions that should be incorporated into a revised document.

Please submit your revised manuscript by Aug 15 2020 11:59PM. If you will need more time than this to complete your revisions, please reply to this message or contact the journal office at plosone@plos.org. When you're ready to submit your revision, log on to https://www.editorialmanager.com/pone/ and select the 'Submissions Needing Revision' folder to locate your manuscript file.

Please include the following items when submitting your revised manuscript:

- A rebuttal letter that responds to each point raised by the academic editor and reviewer(s). You should upload this letter as a separate file labeled 'Response to Reviewers'.
- A marked-up copy of your manuscript that highlights changes made to the original version. You should upload this as a separate file labeled 'Revised Manuscript with Track Changes'.
- An unmarked version of your revised paper without tracked changes. You should upload this as a separate file labeled 'Manuscript'.

If you would like to make changes to your financial disclosure, please include your updated statement in your cover letter. Guidelines for resubmitting your figure files are available below the reviewer comments at the end of this letter.

If applicable, we recommend that you deposit your laboratory protocols in protocols.io to enhance the reproducibility of your results. Protocols.io assigns your protocol its own identifier (DOI) so that it can be cited independently in the future. For instructions see: <http://journals.plos.org/plosone/s/submission-guidelines#loc-laboratory-protocols>

We look forward to receiving your revised manuscript.

Kind regards,

Brenda A Wilson, Ph.D.
Academic Editor
PLOS ONE


[Note: HTML markup is below. Please do not edit.]

Reviewers' comments:

Reviewer's Responses to Questions

**Comments to the Author**

1. If the authors have adequately addressed your comments raised in a previous round of review and you feel that this manuscript is now acceptable for publication, you may indicate that here to bypass the “Comments to the Author” section, enter your conflict of interest statement in the “Confidential to Editor” section, and submit your "Accept" recommendation.

Reviewer #1: All comments have been addressed

Reviewer #2: (No Response)

Reviewer #3: All comments have been addressed

Reviewer #4: (No Response)

2. Is the manuscript technically sound, and do the data support the conclusions?

The manuscript must describe a technically sound piece of scientific research with data that supports the conclusions. Experiments must have been conducted rigorously, with appropriate controls, replication, and sample sizes. The conclusions must be drawn appropriately based on the data presented.

Reviewer #1: Yes

Reviewer #2: Yes

Reviewer #3: Yes

Reviewer #4: Yes

3. Has the statistical analysis been performed appropriately and rigorously?

Reviewer #1: Yes

Reviewer #2: Yes

Reviewer #3: Yes

Reviewer #4: Yes

4. Have the authors made all data underlying the findings in their manuscript fully available?

The [PLOS Data policy](http://www.plosone.org/static/policies.action#sharing) requires authors to make all data underlying the findings described in their manuscript fully available without restriction, with rare exception (please refer to the Data Availability Statement in the manuscript PDF file). The data should be provided as part of the manuscript or its supporting information, or deposited to a public repository. For example, in addition to summary statistics, the data points behind means, medians and variance measures should be available. If there are restrictions on publicly sharing data—e.g. participant privacy or use of data from a third party—those must be specified.

Reviewer #1: Yes

Reviewer #2: Yes

Reviewer #3: (No Response)

Reviewer #4: Yes

5. Is the manuscript presented in an intelligible fashion and written in standard English?

PLOS ONE does not copyedit accepted manuscripts, so the language in submitted articles must be clear, correct, and unambiguous. Any typographical or grammatical errors should be corrected at revision, so please note any specific errors here.

Reviewer #1: Yes

Reviewer #2: Yes

Reviewer #3: Yes

Reviewer #4: Yes

6. Review Comments to the Author

Please use the space provided to explain your answers to the questions above. You may also include additional comments for the author, including concerns about dual publication, research ethics, or publication ethics. (Please upload your review as an attachment if it exceeds 20,000 characters)

Reviewer #1: The authors are congratulated for their execution and presentation of this important study. All of my previous comments have been satisfactorily addressed. Only 2 minor comments:

**We thank the reviewer for their kind words and for their comments.**

Abstract: Please add a statement describing the relevant results that support the statement “…and its impact on job performance”.

**We have added results to support this statement, and modified the abstract to keep it within the 300 word limit.**

Discussion states “Our results are consistent with these prior observations, as we detect no differences for alpha diversity between TD+ and TD- subjects. It should be emphasized that while our results are statistically significant, the number of independent samples are small (N=4 TD- subjects, N=7 TD+ subjects).” Should the second sentence read “not statistically significant”?

**We thank the reviewer for this change. We agree that for the results described in this paragraph should be changed to ‘not statistically significant’. We also discuss our small sample size when we comment on statistically significant results later in the Discussion.**

Reviewer #2: (No Response)

Reviewer #3: Thank you for the authors for addressing all my comments. the manuscript now is accepted from my side with no further comments or modification.

**We thank the reviewer for their time and effort.**

Reviewer #4: Dear authors,
Thank you for being responsive to my recommendations and for the clarifications. I do not have any major concerns for the revised manuscript. It is much clearer and easier to understand.
Best wishes,
Akemi W.

**We thank you for your comments and suggestions.**

Minor Comments:
Comment 1, Page 2– Suggest revising the following sentence to include explanations for each of the abbreviations that are mentioned for the first time.
Suggested revision: “We conducted a passive surveillance study of all cases of diarrhea reporting to the medical unit with 152 total cases and a similar pattern of etiology with 52/152 enteroaggregative E. coli (EAEC), 50/152 enteroinvasive E. coli (EIEC), and 35/152 enteropathogenic E. coli (EPEC) as the most prevalent pathogens detected, and ……”

**We have added explanations for each of the abbreviations as suggested by the reviewer, and modified the abstract to keep it within the 300 word limit.**

Introduction-
Comment 2, Page 5- Evidence related to microbiome and TD is not sufficient, the research gap not identified, and the hypothesis is still not specific for the current research.
Of note, David et al, 2015 was the only study cited. Please consider including additional literature evidence on the relationship between microbiome and TD

**We thank the reviewer for these inputs.**

**We have added additional literature references supporting a relationship between the gut microbiome and TD. We delve into these further in the Discussion where we compare these papers to our results, and so did not feel that we should repeat an in-depth summary of the literature here, other than providing a broad summary of their results.**

**We have made the hypothesis specific for the current research by adding ‘can contribute to susceptibility or resilience to TD’ to the final sentence of the paragraph.**

**We have further identified the research gap at the beginning of the next paragraph by restating why a better understanding of how microbial community composition affects military personnel experiencing diarrhea during deployment is important.**

Comment 3, Page 5- recommend clarifying what “illness” is in the manuscript.
Suggested revision: “After enrollment and collection of baseline samples, subjects were contacted weekly to assess the presence of any ailment specifically diarrheal diseases and if ill, ….”

**We have added clarification based on the reviewers suggestion.**

Discussion-
Comment 4-Should it be just recall bias?
Suggested revision: “Similarly, questionnaires performed at the end of a person’s deployment may not accurately capture what they experienced because of recall bias.”

**Yes, we agree. We changed the sentence to the reviewer’s suggestion.**

7. PLOS authors have the option to publish the peer review history of their article ([what does this mean?](https://journals.plos.org/plosone/s/editorial-and-peer-review-process#loc-peer-review-history)). If published, this will include your full peer review and any attached files.


If you choose “no”, your identity will remain anonymous but your review may still be made public.


**Do you want your identity to be public for this peer review?** For information about this choice, including consent withdrawal, please see our [Privacy Policy](https://www.plos.org/privacy-policy).

Reviewer #1: No

Reviewer #2: No

Reviewer #3: No

Reviewer #4: **Yes:** Akemi T Wijayabahu

[NOTE: If reviewer comments were submitted as an attachment file, they will be attached to this email and accessible via the submission site. Please log into your account, locate the manuscript record, and check for the action link "View Attachments". If this link does not appear, there are no attachment files.]

While revising your submission, please upload your figure files to the Preflight Analysis and Conversion Engine (PACE) digital diagnostic tool, <https://pacev2.apexcovantage.com/>. PACE helps ensure that figures meet PLOS requirements. To use PACE, you must first register as a user. Registration is free. Then, login and navigate to the UPLOAD tab, where you will find detailed instructions on how to use the tool. If you encounter any issues or have any questions when using PACE, please email PLOS at [figures@plos.org](mailto:figures@plos.org). Please note that Supporting Information files do not need this step.

*In compliance with data protection regulations, you may request that we remove your personal registration details at any time.*  [*(Remove my information/details)*](https://www.editorialmanager.com/pone/login.asp?a=r)*. Please contact the publication office if you have any questions.*
